# Lipidomic signatures in Colombian adults with metabolic syndrome

**DOI:** 10.1007/s40200-024-01423-5

**Published:** 2024-05-04

**Authors:** María Fernanda Serna, Milton Fabián Suarez-Ortegón, Eliécer Jiménez-Charris, Isabella Echeverri, Mónica P. Cala, Mildrey Mosquera

**Affiliations:** 1 Grupo de Nutrición, Departamento de Ciencias Fisiológicas, Facultad de Salud, Universidad del Valle, Calle 4B #36-00 Cali, Colombia; 2 Departamento de Alimentación y Nutrición, Facultad de Ciencias de La Salud, Pontificia Universidad Javeriana Seccional Cali, Colombia. Cl. 18 #118-250, Barrio Pance, 760031 Cali, Valle del Cauca Colombia; 3 Faculty of Health Sciences, Universidad Icesi, Cali, Colombia; 4 Metabolomics Core Facility-MetCore, Vice Presidency for Research, Universidad de los Andes, Carrera 1, #18A-12 Bogotá, Colombia

**Keywords:** Metabolic Syndrome, Lipidomics analysis, Insulin resistance, Abdominal obesity, Metabolome

## Abstract

**Background and Aims:**

Metabolic syndrome (MetS) comprises a set of risk factors that contribute to the development of chronic and cardiovascular diseases, increasing the mortality rate. Altered lipid metabolism is associated with the development of metabolic disorders such as insulin resistance, obesity, atherosclerosis, and metabolic syndrome; however, there is a lack of knowledge about lipids compounds and the lipidic pathways associated with this condition, particularly in the Latin-American population. Innovative approaches, such as lipidomic analysis, facilitate the identification of lipid species related to these risk factors. This study aimed to assess the plasma lipidome in subjects with MetS.

**Methods:**

This correlation study included healthy adults and adults with MetS. Blood samples were analyzed. The lipidomic profile was determined using an Agilent Technologies 1260 liquid chromatography system coupled to a Q-TOF 6545 quadrupole mass analyzer with electrospray ionization. The main differences were determined between the groups.

**Results:**

The analyses reveal a distinct lipidomic profile between healthy adults and those with MetS, including increased concentrations of most identified glycerolipids -both triglycerides and diglycerides- and decreased levels of ether lipids and sphingolipids, especially sphingomyelins, in MetS subjects. Association between high triglycerides, waist circumference, and most differentially expressed lipids were found.

**Conclusion:**

Our results demonstrate dysregulation of lipid metabolism in subjects with Mets, supporting the potential utility of plasma lipidome analysis for a deeper understanding of MetS pathophysiology.

**Supplementary Information:**

The online version contains supplementary material available at 10.1007/s40200-024-01423-5.

## Introduction

Metabolic syndrome (MetS) is a collection of risk factors associated with the development of chronic and cardiovascular diseases. Individuals with MetS face a fivefold higher risk of developing type 2 diabetes and a three times higher risk of a heart attack or stroke [1]. The diagnosis of MetS involves three to five risk components, including dysregulation of blood pressure, central obesity, dyslipidemia, and impaired glucose metabolism [2]. The presence of even one to two components of MetS has been correlated with an increase in mortality [3], and the prevalence of MetS is estimated to be greater than 20% for the North American and Latin American populations [4, 5].

MetS components have been linked, through metabolomic studies, to over 300 metabolites found in biological fluids, primarily associated with altered glucose levels and lipids [6]. Changes in the metabolism of fatty acids and triglycerides appear to involve mechanisms inducing insulin resistance and a pro-inflammatory state. This occurs through the excessive accumulation of free fatty acids in the liver and muscle, along with the secretion of cytokines that are overexpressed in obesity [7, 8]. Furthermore, imbalances in the lipid signaling pathways controlling various cellular processes have been implicated in the development of pathological conditions such as MetS through signaling lipids such as sphingolipids, fatty acids, and eicosanoids [9].

The analysis and identification of lipids through liquid chromatography coupled with mass spectrometry (LC–MS) have been instrumental in establishing associations between lipid species and cardiometabolic risk. Multiple lipids have been linked to conditions such as obesity, hypertension, insulin resistance, type 2 diabetes, and MetS [10–12]. Moreover, lipidomic analysis of blood has unveiled alterations in several lipid species associated with metabolic diseases and the discovery of new lipids distinguishing normal conditions from disease states [13].

Studies have documented distinct metabolomic and lipidomic profiles associated with MetS and its components. Using lipidomic approaches, these studies have revealed different patterns of lipid alterations in individuals with MetS compared to healthy controls [6]. This analysis provides valuable insights into the specific lipid alterations associated with MetS, highlighting potential biomarkers for early detection and therapeutic targets for intervention. However, there is a lack of information concerning the Latin-American population, particularly in individuals without another associated disease. Understanding these lipidomic signatures is important for developing region-specific diagnostic tools and personalized treatment strategies for MetS in Latin America. Consequently, we aimed to identify the variations in lipidomic profiles and metabolic pathways between individuals with and without MetS by a nondirected lipidomics analysis to provide valuable insights into the metabolic pathways of MetS in the Latin-American population.

## Materials and methods

### Characterization of the subjects studied and sample collection

The present study utilized samples from a previous investigation conducted by Suarez-Ortegon et al. [14] to explore iron levels and their association with insulin sensitivity and type 2 diabetes in adults. The original study, conducted between 2009 and 2010, involved 245 adult subjects of both sexes who did not have systemic diseases (except for type I obesity (body mass index (BMI) ≥ 30 and < 35)), chronic communicable diseases, or infectious or inflammatory processes at the time of enrollment. This study adhered to the guidelines outlined in the Declaration of Helsinki's guidelines, received approval from the ethics committee of the University of Valle, and obtained informed consent from all participants. Additionally, participants of the study granted authorization to use their data in subsequent research.

For the present study, we selected a subpopulation of 80 subjects with serum samples stored at -80 °C in the Nutrition group´s sample bank. These samples were divided into two groups: **1. Healthy (control) group (n = 40) (HeS)** consisting of adults without systemic or inflammatory diseases and lacking risk factors or characteristics of MetS; **2. MetS group (n = 40)** included adults who met 3–5 criteria for diagnosing MetS. The cases of MetS were identified based on the criteria of the Harmonized definition of MetS (2), which includes: a. Elevated waist circumference: men ≥ 90 cm and women ≥ 80 cm; b. High triglycerides: ≥ 150 mg/dL (1.7 mmol/L), c. Reduced high-density lipoprotein cholesterol (HDL-C): < 40 mg/dL (1.0 mmol/L) in men; < 50 mg/dL (1.3 mmol/L) in women; d. Elevated blood pressure: systolic ≥ 130 and/or diastolic ≥ 85 mmHg, e. Elevated fasting glucose: ≥ 100 mg/dL.

The participant database and relevant anthropometric and clinical data were extracted from the previous study. Subsequently, samples were obtained from the sample bank of the Nutrition group at the Universidad del Valle in Cali, Colombia. After selecting samples for the current study, they were appropriately labeled, refrigerated, and transported to the metabolomics laboratory of the University of the Andes, MetCore, in Bogotá, Colombia, for processing.

### Anthropometric and clinical information

The patients underwent a 12-h fasting period for blood sampling, blood pressure measurement and anthropometric assessment. Personal and family data was gathered through a survey. At the same time, waist circumference (WC) was measured with a metric band, height with a stadiometer, weight on a digital scale, and body fat percentage using a portable impedance meter (OMRON®). BMI was calculated by the formula weight/(height^2^). Systolic and diastolic pressures were measured twice using a digital sphygmomanometer (OMRON®) in the supine position in the right arm with an interval of 10 min between the two measurements. Blood samples were collected via antecubital vein puncture, with 15 mL of blood extracted into three Vacutainer® tubes without additive. Samples were transported to the laboratory in refrigerators between 4–8 °C, and within an hour, they were centrifuged at 3000 rpm to obtain serum, which was then stored at -20 °C until processing. Biochemical markers were analyzed: high-sensitivity C-reactive Protein using turbidimetry; glucose, total cholesterol, triglycerides, and HDL-C using enzymatic colorimetric assays (BioSystems SA®) and insulin by chemiluminescence (Immulite®). The homeostatic model assessment of insulin resistance (HOMA-IR) index [15] was calculated with the following formula:

$$HOMA-IR= \frac{Fasting glucose \left(\frac{mmol}{L}\right)\times Fasting insulin (\frac{mU}{L})}{22.5}$$

### Lipidomic analysis

For lipid extraction, 50 μL of serum was mixed with a combination of 175 μL of MeOH at -20 °C and 175 μL of MTBE at room temperature, vortexed at 3200 rpm for 15 min, centrifuged at 16,000 g at 24 °C for 10 min. Finally, the supernatant (upper phase containing the lipids) was taken, and 80 μL was transferred to an LC–MS vial for subsequent analysis. The lipidomic profile was determined using an Agilent Technologies 1260 liquid chromatography system coupled to a Q-TOF 6545 quadrupole mass analyzer with electrospray ionization. Five microliters of the sample extract were injected into a C18 column (InfinityLab Poroshell 120 EC-C18 (150 × 3.0 mm, 2.7 μm)). Liquid chromatography separation was performed at 60 °C using a mobile phase consisting of 10 mM of formate and 0.1% formic acid in ACN-H_2_O (60:40) (Phase A) and 10 mM formate and 0.1% formic acid in IPA-ACN (90:10) (Phase B) with a flow constant of 0.5 mL/min. The elution gradient varied from 15 to 99% B in 11.5. The gradient was maintained for 0.5 min at 99% before the gradient returned to its initial conditions and was kept constant for 6 min to ensure re-equilibration of the column. The data were collected in positive and negative ESI ionization modes in separate runs, operated in full scan mode from 100 to 1700 m*/z*. Throughout the analysis, two masses of reference: *m/z* 121.0509 (C_5_H_4_N_4_) and *m/z* 922.0098 (C_18_H_18_O_6_N_3_P_3_F_24_) for the positive mode of ionization and *m/z* 112.9856 (C_2_O_2_F_3_(NH_4_)) and *m/z* 1033.9881 (C_18_H_18_O_6_N_3_P_3_F_24_) for the negative ionization mode. Five iterative-MS/MS runs were performed using a QC sample for both ion modes at the end of the analytical run. The iterative-MS/MS runs were set with a collision energy of 20 and 40 eV.

### Quality control samples

The quality control (QC) samples were prepared by mixing equal volumes of each sample. First, the QC samples were analyzed following the procedures described above. Then, several QC runs were performed to determine the reproducibility of the sample preparation and the stability of the analytical platform used until the analytical system was equilibrated. After that, the QC samples were analyzed for every 10 randomized samples.

### Data processing and analysis

The data are presented as the mean ± standard deviation for the subjects' characteristics. Statistical analyses were performed using IBM SPSS 25.0. The differences between the two groups were analyzed using the independent t-test (parametric distribution) for means with continuous data and the chi-squared test for categorical data. A value of *p* < *0.05* was defined as statistically significant.

For lipidomic analysis, the significant differences between the serum samples of both groups were evaluated by univariate (UVA) and multivariate statistical analysis (MVA). The UVA was performed using MATLAB (7.10.0 MathWorks, Inc., Natick), and the MVA was performed employing MatLab (7.10.0 Mathworks, Inc., Natick). First, data normality was verified by evaluating the Kolmogorov–Smirnov-Lillefors and Shapiro—Wilk tests and the variance ratio by Levene's test. Next, the p-value was determined by parametric (unpaired t-test) or non-parametric (Mann—Whitney U test) tests with a Benjamini—Hochberg False Discovery Rate posthoc correction (FDR). MVA was performed using SIMCA-P + 16.0 software (Umetrics, Umea, Sweden), in which an unsupervised principal component analysis (PCA) was performed to observe the unsupervised distribution of the analyzed samples. Then, a supervised orthogonal projection to latent structures discriminant analysis (OPLS-DA) model was performed to select the molecular characteristics responsible for separating the groups (HeS group and Mets group). For this comparison, the statistically significant molecular markers were chosen with the following requirements: 1) UVA: *p* < *0.05* and 2) MVA: variance important in projection (VIP) > 1 with jack-knife confidence interval (JK) not included 0.

To compare the effect of the components related to MetS to the explanation of the intersubject variability, one-way analysis of variance (ANOVA) and analysis of covariance were performed to determine the significant differences between the components of the MetS and the lipids identified adjusted for age and sex as covariates, a corrected *p* < *0.0004* was considered statistically significant for this analysis. The correlations of the latter with obesity, BMI, insulin, and HOMA-IR were also determined. A corrected *p* < *0.002* was considered statistically significant for this analysis. All p values obtained were corrected for multiple comparisons using the Bonferroni fit test [16, 17].

### Lipid annotation

The lipids obtained in the LC–MS analysis were identified with three identification tools: molecular formula generator, available online databases using the CEU mass mediator tool, and MS/MS analysis. First, the statistically significant molecular features were tentatively identified based on the MS1 data using our online tool CEU Mass Mediator (http://ceumass.eps.uspceu.es/mediator/) through a search in METLIN databases (http://metlin.scripps.edu), KEGG (http://genome.jp/kegg), lipid MAPS (http://lipidMAPS.org), and HMDB (http://hmdb.ca). The tentative assignment was performed based on accurate mass with a maximum mass error tolerance of 10 ppm, the isotopic pattern distribution in the molecular formula generated in the experimental data, the possibility of cation and anion formation and adduct formation, and the retention time. Then, a manual MS/MS spectral interpretation was carried out comparing MS/MS fragmentation to the available spectra data in MS-DIAL, LIPID MAPS, and the Lipid Annotator software (Agilent Technologies Inc., Santa Clara, CA, USA). Finally, the identification level was assigned according to Blaženović, I. et al. [18].

### Pathway analysis

The metabolic pathways were analyzed using the MetaboAnalyst 5.0 tool (http://www.metaboanalyst.ca/), which integrates two approaches: pathway enrichment analysis and pathway topology analysis. In addition, a list of compound names of the identified significant lipids was included and processed using the "*Homo sapiens*" library. [19]. The selected metabolic pathways had a higher impact on the analysis.

## Results

Table 1 presents the characteristics of the study participants, comprising 40 individuals with MetS and 40 without MetS. No significant differences in age and sex were observed, although there was a higher proportion of women in healthy subjects’ group. Following diagnostic criteria, participants with MetS exhibited higher values in blood pressure, waist circumference, BMI, insulin, glucose, triglycerides (TG), HOMA-IR, high-sensitivity C-reactive protein, and lower HDL cholesterol than the control group. However, the two groups had no significant differences in low-density lipoprotein (LDL) or cholesterol.

**Table 1:** Clinical characteristics of the population

Variables	Metabolic syndrome—MetS (n = 40)	Healthy Subjects – HeS (n = 40)	*p-value*
Age (years)	47.98 ± 8.021	44.28 ± 8.569	0.050
Women n (%)	13 (32.5%)	21 (52.5%)	0.113
BMI	28.92 ± 3.39	23. 34 ± 3.45	< 0.001
Systolic blood pressure (mmHg)	132.9 ± 14.63	109.575 ± 12.14	< 0.001
Diastolic blood pressure (mmHg)	82.65 ± 8.54	70.15 ± 9.42	< 0.001
Waist circumference (cm)	89.78 ± 9.95	71.58 ± 7.05	< 0.001
Insulin	15.34 ± 6.63	5.95 ± 2.53	< 0.001
HDL mg/dL	40. 7 ± 6.92	56. 99 ± 9.78	< 0.001
LDL mg/dL	114. 57 ± 27. 0	125.67 ± 29. 16	0.088
Cholesterol mg/dL	203. 95 ± 33.62	201.83 ± 33.42	0.778
CRP-hs mg/dL	2.89 ± 3.0	1.48 ± 0.80	0.005
Glucose mg/dL	95. 45 ± 14. 80	85. 30 ± 6.24	< 0.001
TG mg/dL	249.18 ± 114.77	95.98 ± 30.38	< 0.001
VLDL	45.79 ± 18.36	19.24 ± 6.1	< 0.001
HOMA-IR	3.48 ± 1.76	1.18 ± 0.52	< 0.001

Values are the mean ± standard deviation or percentage; p-value < 0.05; TG (triglycerides); BMI (body mass index); CRP-hs (High sensitivity C-reactive protein); HDL (high-density lipoprotein); LDL (low-density lipoprotein).

**Fig. 1:**
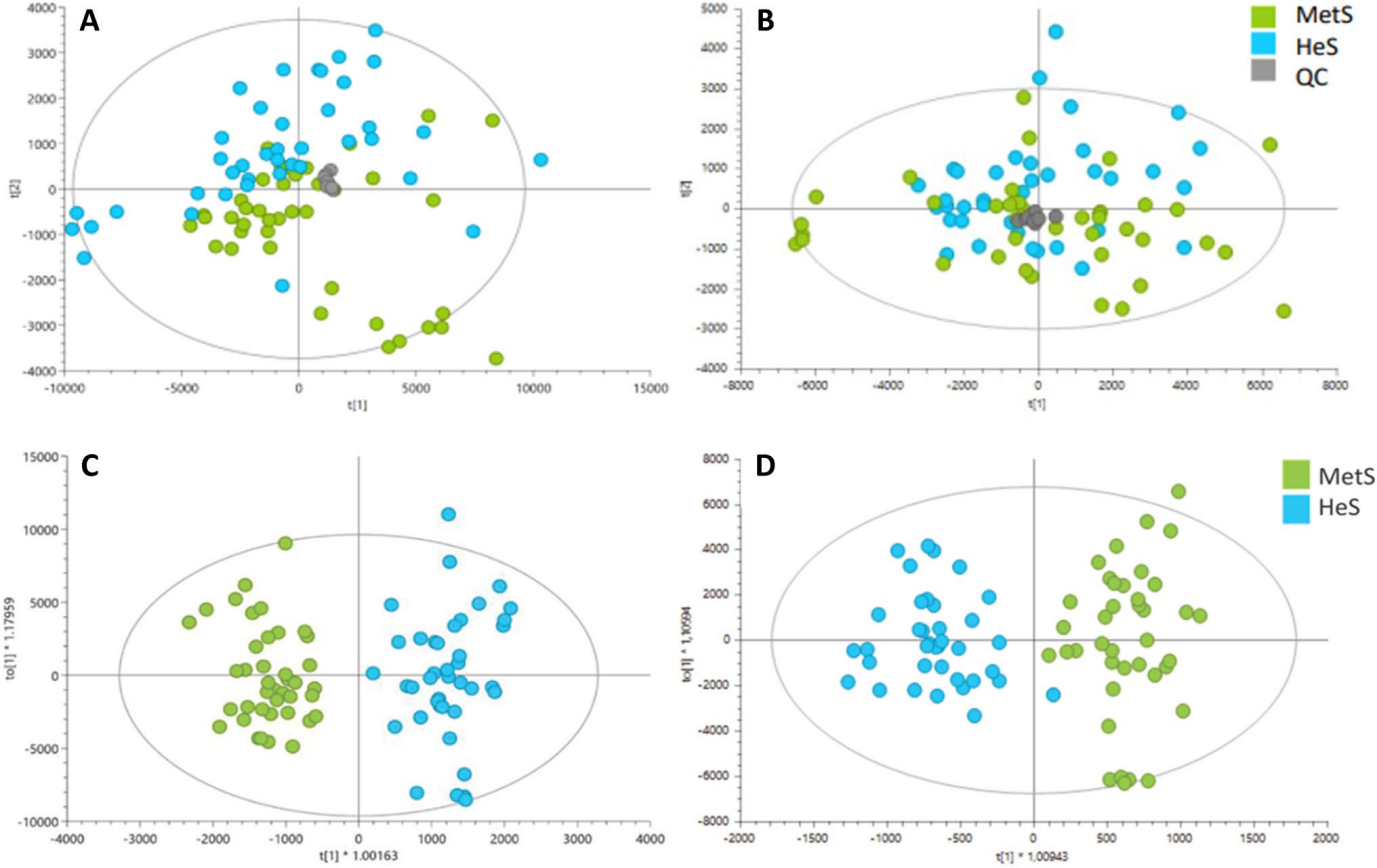
PCA score plots for global lipidomics of both polarities. (MetS: blue points,HeS: green points), quality control samples: gray dots) A. LC-QTOF-MS ( +): R^2^ (cum):0.901, Q^2^ (cum): 0.703; B. LC-QTOF-MS (-): R^2 ^(cum): 0.896, Q^2^ (cum): 0.6227. OPLS-DA MODELS. Lipidomics of the MetS (blue points) and HeS (green points) groups. **C.** OPLS-DA in ESI + , LC-QTOF-MS ( +): R^2^_(cum)_:0.804, Q^2^_(cum)_: 0.681, cv-anova: 1.99e-12; **D** OPLS-DA in ESI-, LC-QTOF-MS(-): R^2^_(cum)_: 0.707, Q.^2^_(cum)_: 0.609, cv-anova: 5.211e-9

The reproducibility of the analytical platforms was assessed by grouping the quality control (QC) samples through Principal Component Analysis (PCA), ensuring data quality and the preservation of the biological variation across the experimental data (Fig. 1). The PCA model exhibited a tendency to separate the samples from healthy subjects and those with MetS in the score plot.

Both unsupervised (PCA) and supervised statistical analyses (OPLS-DA) demonstrated a distinct separation between the groups of healthy subjects (depicted as green points) and the subjects with MetS (represented as blue points). Clear separations were achieved from the orthogonal predictive components of the OPLS-DA model between the two groups, with acceptable values of predictive variance (R^2^) (LC-QTOF-MS ( +): R^2^_(cum)_:0.804, LC-QTOF-MS(-): R^2^_(cum)_: 0.707), predictive ability (Q^2^) (LC-QTOF-MS ( +): Q^2^_(cum)_: 0.681; LC-QTOF-MS(-): Q^2^_(cum)_: 0.609) and *p-value* in the CV-ANOVA tests (LC-QTOF-MS ( +): cv-ANOVA: 1.99e-12; LC-QTOF-MS(-): cv-ANOVA: 5.211e-9) indicating that the OPLS-DA models are not overfitting (Fig. 1).

A total of 100 lipids exhibited statistical differences between MetS and HeS through the MS-based platforms: 12 lipids by LC–MS (-) (Table S1) and 88 lipids by LC–MS ( +) (Table S2). The significant lipids belonged to various classes, including glycerolipids (di-acylglycerols (DG) and triglyceride (TG)); glycerophospholipids (lysophosphatidylcholines (LPC), phosphatidylcholine (PC) and ether phospholipids); and sphingolipids (sphingomyelins (SM). Glycerolipids showed increased levels in the MetS group, whereas glycerophospholipids and sphingolipids exhibited decreased in the MetS group compared to HeS group. After adjusting for sex and age, nineteen lipids showed distinct expression patterns between MetS and the HeS group (Table 2).

**Table 2:** Lipids identified with differential expression in subjects with or without MetS using LC–MS ( ±)

								MetS vs. HeS		
Compound	Formula	Mass	RT (min)	Mass Error (ppm)	Adduct	^a^ CV for QC (%)	^b^ CON	^c^ Change (%)	^d^ VIP	^*e*^*p* value
*Glycerolipids*										
DG 36:5	C_39_H_66_O_5_	614.491	10.93	2	M + H	9.4	Putative	65	1.00568	7.72E-07*
DG 36:3	C_39_H_70_O_5_	618.5223	10.99	2	M + NH_4_	12.9	MS/MS	46	1.08707	0.0142
DG 34:1	C_37_H_70_O_5_	594.5223	11.54	2	M + H	6.4	Putative	114	2.10361	1.30E-07*
DG 34:2	C_39_H_72_O_5_	620.538	11.58	2	M + NH_4_	16.7	Putative	74	1.11505	3.02E-05*
DG 38:5	C_41_H_70_O_5_	642.5223	11.58	1	M + H	18	Putative	105	1.13776	1.01E-08*
TG 61:14	C_64_H_96_O_6_	960.7207	11.4	5	M + CHO_2_^−^	3.4	Putative	72	1.49631	0.019*
DG 42:6	C_45_H_76_O_5_	696.5693	11.88	2	M + NH_4_	5.3	Putative	135	1.20987	5.36E-10*
*Glycerophospholipids*										
LPC 18:2	C_26_H_50_NO_7_P	519.3325	2.76	2	M + H	4.7	MS/MS	-39	1.04453	0.0122
LPC 20:4	C_28_H_50_NO_7_P	543.3325	2.96	2	M + H	7.5	MS/MS	-41	1.59858	0.0212
LPC 18:1	C_26_H_52_NO_7_P	521.3481	4.18	3	M + H	4	MS/MS	-31	2.15157	0.0397
PC O-34:3 // PC P-34:2	C_42_H_80_NO_7_P	741.5672	9.7	1	M + H	2.6	MS/MS	-39	1.81418	2.12E-05*
PE O-37:2 // PE P-37:1	C_42_H_82_NO_7_P	743.5829	9.85	1	M + H	4.4	MS/MS	-38	1.25677	0.0001
PC 33:0	C_41_H_80_NO_8_P	745.5622	10.47	2	M + H	2.8	MS/MS	-31	1.07478	0.0001*
PC P-33:2 // PC O-33:3	C_41_H_78_NO_7_P	727.5516	10.8	2	M + H	10.7	Putative	-35	3.3864	0.0001*
*Sphingolipids*										
SM 34:0;O2	C_39_H_81_N_2_O_6_P	704.5832	9.37	1	M + H	2.6	MS/MS	-30	1.05003	0.0026
SM 40:2;O2	C_45_H_89_N_2_O_6_P	784.6458	10.85	2	M + H	15.2	MS/MS	-33	2.48024	0.0004
SM 41:2;O2	C_46_H_91_N_2_O_6_P	798.6615	11.09	1	M + H	9.8	MS/MS	-33	1.32051	0.0002*
SM 39:1;O2	C_44_H_89_N_2_O_6_P	772.6458	11.15	1	M + H	7.7	MS/MS	-33	1.68548	0.00044*
SM 41:1;O2	C_46_H_91_N_2_O_6_P	798.6615	11.24	2	M + H	9.2	Putative	-40	2.60912	1.26E-05*

^a^ CV for QC (%): CV obtained for the same compound within the set of quality control samples. ^b^ CON: lipid confirmation by MS/MS. ^c^ Change: percentage change in abundances, calculated as MetS/HeS. The sign indicates the change in direction. ^d^ VIP: values with estimation of Jack-Knife confidence interval without including the 0—confidence level: 95%. ^e^ p values corrected by Benjamin Hochberg (FDR correction); * significant lipids after adjustment for sex and age covariates. Abbreviations: DG: Diacylglycerol, TG: Triglycerides, LPC: Lysophosphatidylcholines, PC: Phosphatidylcholine, PE: Phosphatidylethanolamine, SM: Sphingomyelin

Furthermore, we sought to assess if any of the 100 identified lipids exhibited individual correlations with components of Metabolic Syndrome. Our analysis revealed that thirty-nine lipids were identified as correlated with one or more MetS components (Table S3). Additionally, to investigate if variations in lipid expression related to MetS components persisted after accounting for age and sex differences between groups, we conducted an adjustment. The results showed that 18 lipids continued to exhibit differential expression between groups, even after adjusting for age and sex.

Table 3 presents the list of lipids associated with each MetS component. Among the 18 lipids linked to individual components of MetS, eleven glycerolipids notably contributed to the differentiation between the groups (VIP > 1). Notably, this analysis revealed that ninety-five percent of the lipids associated with MetS components were linked to the triglyceride (TG) component, and all identified TGs had higher expression in subjects with MetS. The waist circumference component showed associations with ten identified lipids, nine exhibiting higher expression in subjects with MetS than HeS. In comparison, one lipid (PC O-44:5 // PC P-44:4) showed lower expression in the MetS group. The HDL and systolic blood pressure components were associated with three and two lipids, all of which had higher expression in subjects with MetS compared to healthy controls.

**Table 3:** Lipids with differential expression between metabolic syndrome components identified by ANCOVA

Lipid class	Lipid	^a^ Change	^b^ VIP	TG	^c^ Waist	HDL	SBP
Glycerolipids	DG 38:7	35	-	3.08E-04	-	-	-
	DG 32:1	143	-	2.04E-07	4.10E-04	-	-
	DG 36:5	65	1.00568	1.37E-08	4.60E-05	-	-
	DG 36:3	46	1.08707	2.30E-05	-	-	-
	DG 38:6	74	-	1.20E-04	-	-	-
	DG 36:2	64	-	1.81E-06	-	4.12E-04	
	DG 34:3	62	-	4.04E-08	2.46E-07	-	-
	DG 34:1	114	2.10361	5.89E-09	1.27E-05	-	-
	DG 34:2	74	1.11505	4.16E-07	4.74E-04	1.30E-04	-
	DG 38:5	105	1.13776	1.84E-11	5.49E-06	2.99E-07	-
	DG 42:6	135	1.20987	9.57E-07	1.68E-04	-	8.53E-05
Glycerophospholipids	PI 32:1	57	-	3.58E-04	-	-	-
	PI 40:6	21	-	2.06E-04	-	-	-
	PA P-34:3 // PA O-34:4	43	-	7.68E-05	-	-	-
	PC 33:2	129	-	1.12E-06	4.58E-04	-	
	PA P-32:2 // PA O-32:3	100	-	1.01E-08	3.85E-05	-	9.09E-05
	PC 33:1	42	-	1.97E-05	-	-	-
	PA P-34:2 // PA O-34:3	131	-	2.68E-07	-	-	-
	PC O-44:5 // PC P-44:4	-28	-	-	4.66E-04	-	-

The values expressed in the table correspond to the p values corrected by Bonferroni correction for each lipid concerning the components of the metabolic syndrome obtained by the ANCOVA test and adjusted for age and sex covariates. ^a^ Change: percentage of change in abundances, calculated as MetS/control, the sign indicates the direction of change. ^b^ VIP: values with estimation of Jack-Knife confidence interval without including the 0—confidence level: 95%. Abbreviations: HDL: High-Density Lipoproteins, TG: triglycerides, ^c^ Waist: Waist circumference, SBP: Systolic blood pressure, DG: Diacylglycerol, TG: Triglycerides, PI: Phosphatidylinositol, PA: Phosphatidic acid, PC: Phosphatidylcholine.

Given the well-established role of obesity and insulin resistance in Metabolic Syndrome development, we calculated correlations between the 18 lipids exhibiting differential expression and key MetS components: BMI, insulin, and HOMA-IR. This analysis revealed nine lipids positively correlated with BMI (Table 4), 13 with insulin, and 14 with HOMA-IR. Notably, PC O-44:5 // PC P-44:4 demonstrated a negative correlation with insulin and HOMA-IR (Table 5). All lipids associated with BMI, insulin, and HOMA-IR displayed increased levels in the MetS group, except for PC O-44:5 // PC P-44:4, which exhibited a decrease.

**Table 4:** Correlation of lipids with BMI

Lipid	BMI	
	*r*	p-value*
DG 32:1	0.431348	0.000081
DG 36:5	0.383297	0.000533
DG 34:3	0.426851	0.000097
DG 34:1	0.413539	0.000168
DG 34:2	0.344714	0.001998
DG 38:5	0.398038	0.000307
PI 32:1	0.419898	0.000144
PC 33:2	0.388516	0.00044
PA P-32:2 // PA O-32:3	0.353219	0.001513

*p-value < 0.002; r: Pearson coefficient. DG: Diacylglycerol, PI: Phosphatidylinositol, PC: Phosphatidylcholine, PA: Phosphatidic acid

**Table 5:** Lipids correlated with insulin and HOMA-IR

Lipid	Insulin		HOMA-IR	
	*r*	p-value*	*r*	p-value*
DG 32:1	0.516686	9.28E-07	0.45764	0.00002
DG 36:5	0.491206	0.000004	0.420105	0.000105
DG 36:3	0.389898	0.00035	0.330394	0.002761
DG 38:6	0.368394	0.000773	0.297891	0.007281
DG 34:3	0.575852	2.29E-08	0.561106	6.17E-08
DG 34:1	0.532126	3.78E-07	0.483236	0.000006
DG 34:2	0.444025	0.000037	0.392402	0.000318
DG 36:2	0.389442	0.000356	0.335782	0.002327
DG 38:5	0.510663	0.000001	0.460721	0.000017
PI 32:1	0.401736	0.000243	0.395999	0.000303
PC 33:2	0.543563	1.89E-07	0.493441	0.000003
PA P-32:2 // PA O-32:3	0.476268	0.000008	0.41485	0.00013
PC 33:1	0.417919	0.000115	0.377761	0.000551
PC O-44:5 // PC P-44:4	-0.361709	0.000978	-0.370592	0.000715

*p-value < 0.002; r: Pearson coefficient. DG: Diacylglycerol, PI: Phosphatidylinositol, PC: Phosphatidylcholine, PA: Phosphatidic acid, 11

Several lipid species, including DG (34:2), DG (38:5), and phosphatidic acid (PA) (P-32:2), demonstrated associations with at least three MetS components, including BMI, insulin, and HOMA-IR. However, DG (42:6), while associated with three MetS components, did not show correlations with BMI, insulin, or HOMA-IR.

**Fig. 2:**
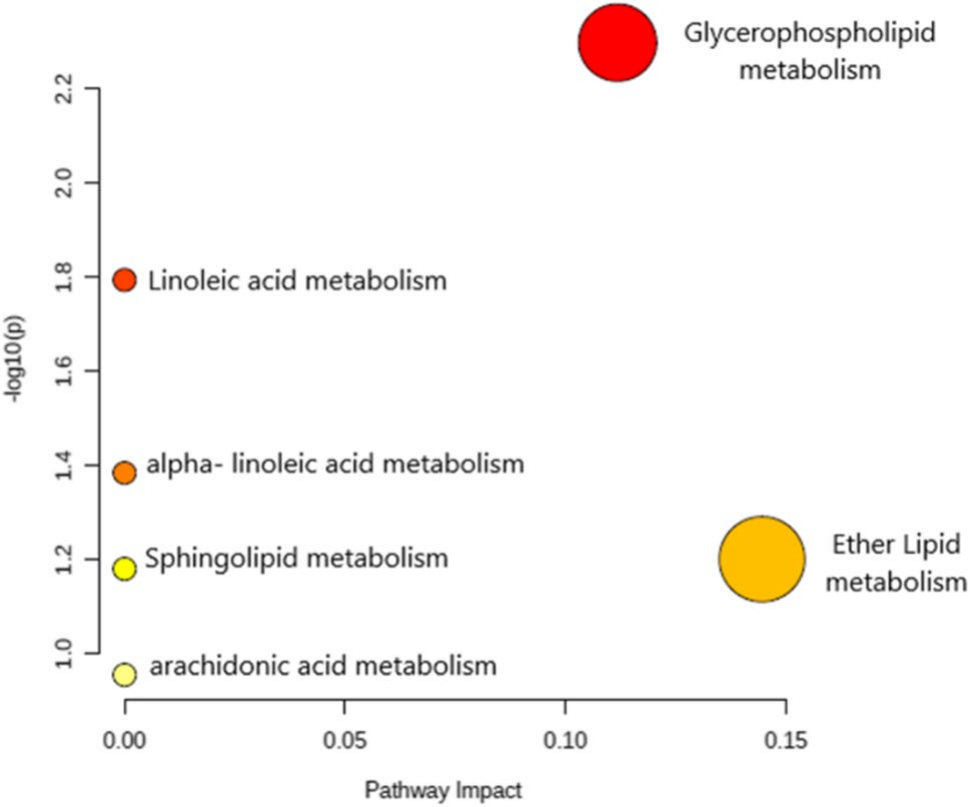
Lipid pathways analysis. Metabolic pathways ordered by pathway enrichment (y-axis) and topology analysis (x-axis) scores are shown using the MetaboAnalyst 5.0 tool. The color and size of each circle are based on p-values and road impact values, respectively

Pathway analysis revealed that the metabolic pathways of glycerophospholipids and lipid ether metabolism were the most significantly affected, showing the highest significance and the high number of identified lipids related to these pathways. Additionally, alterations in the linoleic acid, alpha-linoleic acid, arachidonic acid, and sphingolipid pathways were observed, with fewer lipids identified (Fig. 2).

## Discussion

This study revealed a distinct differentiation in the lipidomic profile between healthy adults and those with MetS. The observed differential expression of the identified lipids aligns with findings from prior research [6, 20]. Specifically, an increase in the expression of most glycerolipids, including triglycerides (TG) and diacylglycerols (DG), was consistent, while the behavior of glycerophospholipids exhibited variability [11, 21, 22].

Notably, most studies indicate a positive association between sphingolipid levels, such as ceramides and sphingomyelins and cardiovascular and metabolic diseases [23, 24]. However, in contrast to these findings, our study observed a decreased expression of sphingomyelins (SMs) in subjects with MetS.

Variability of sphingolipids levels concerning cardiometabolic diseases has been previously documented [25]. Elevated levels of SMs have been identified in obese young individuals and strongly correlated with parameters such as obesity, insulin resistance, liver function, and lipid metabolism [26]. However, reduced serum concentrations of glycerophospholipids and sphingomyelins have also been observed in patients with altered fasting glucose levels or type 2 diabetes compared to healthy controls, even after adjusting for age, sex, and BMI [27]. The variations in SM levels associated with MetS could be related to the fact that SMs can exert based on the composition of their acyl chain. For instance, studies in a murine model of obesity have shown an increase in SM C14 and a decrease in SMs C22, C22:1, and C24 in ob/ob mice compared to lean mice [28]. Other studies have correlated increased in insulin secretion with higher SMs C14:0, C22:3, and C24:4 [29], while C18:0, C20:0, C22:0, and C24:0 have been associated with greater BMI and lower insulin sensitivity [26]. In this study, the SMs contained a C24:1 fatty acid chain, exhibiting more pronounced differential expression in subjects with MetS. Notably, only SM 41:1 played a significant role in distinguishing between the groups and showed a correlation with waist circumference. However, it's important to acknowledge that the expression of these SMs might be influenced by significant variables such as sex and age. Their correlation ceased to be significant after adjusting for these covariates.

Most of the lipid species significantly associated with components of MetS in this study demonstrated strong correlations with triglycerides (TG) and waist circumference, which are robustly linked to obesity [30]. The metabolic profile in obesity and diabetes often exhibits a positive association with elevated concentrations of TG and diacylglycerols (DG) in plasma [11, 31]. High levels of DG have been implicated in the pathophysiological mechanisms underlying insulin resistance in obesity, particularly through activating the protein kinase C pathway [32]. Our study observed upregulation of all TG and DG in subjects with MetS. Specifically, DG (32:1), DG (34:1), DG (34:2), and DG (38:5), which positively correlated with BMI, have been reported in previous studies to correlate with obesity and insulin resistance [31]. Notably, DG (38:5) and DG (34:2) maintained their differential expression with three MetS components after adjustment for age and sex, and they showed positive correlations with insulin levels and the HOMA-IR index. These two DG species have been associated with different indices of insulin sensitivity in prior studies [33, 34]. DG (42:6) exhibited the highest percentage change in concentration between MetS and control groups and remained associated with three MetS components after adjusting for covariates. However, none of these components were insulin, HOMA-IR, or BMI. Notably, DG (42:6) has not been previously reported as a relevant lipid in the metabolic disorder population.

Despite Latin American men and women having the smallest waist circumference cut-off compared to their counterparts in the United States (white, black, and Hispanic populations), they exhibit a higher overall risk [35]. The prevalence of Metabolic Syndrome in Latin American countries indicates that the most frequent components of MetS are low HDL cholesterol levels (62.9%) and abdominal obesity (45.8%) [36]. The INTERHEART study observed that abdominal obesity is the most significant risk factor for cardiovascular diseases in Latin America compared to the rest of the world [37]. Colombia, in particular, stands out with a higher prevalence of elevated cholesterol [38] and high triglycerides [38] compared to other Latin American countries. This observation may explain why most lipid species found in this study are associated with waist circumference and triglycerides.

Pathway analysis (Fig. 2) revealed alterations in the metabolic pathways of ether-bound lipids and glycerophospholipids. We observed a subgroup of phospholipids linked to ether bonds that predominantly exhibited lower concentrations in subjects with Metabolic Syndrome than healthy controls. This disparity contributed to the differentiation between the two groups and was correlated with triglycerides, waist circumference, and systolic blood pressure. Notably, PA (P-32:2), PA (P-34:3), and PA (P-34:2-) showed higher concentrations in the MetS group and corresponded to ether-bound forms of phosphatidic acid. Ether lipids, classified as glycerophospholipids derived from peroxisomes, feature a hydrocarbon chain in the sn-1 position of the glycerol skeleton linked by an ether bond, unlike the more common diacylphospholipids that have an ester bond. They are further categorized into plasmanylphospholipids or plasmenylphospholipids (plasmalogens) [39]. Deficiency in ether lipids has been linked to various diseases. The ether-bound form of PA serves as the precursor for more complex forms of ether phospholipids, where the molecule attached to the phosphate group is generally choline or ethanolamine [39].

The presence of phosphatidic acid linked to ether bonds, particularly in the context of Metabolic Syndrome, has not been reported in previous studies. Among the lipids we identified, three showed concentrations positively correlated with triglyceride levels. Notably, PA (P-32:2) was the sole lipid that exhibited correlations with TG concentration and insulin and Homeostatic Model Assessment of Insulin Resistance (HOMA-IR) levels. Phosphatidic acids play crucial roles as signaling molecules and have been implicated as modulators of insulin signaling. Elevated levels of PAs, especially those containing C16:0 acids, as observed in this study, have been associated with alterations in insulin function and contribute to insulin resistance [40].

In the context of metabolic diseases, the levels of lipids bound to ether bonds can exhibit variations. For instance, Graessler et al. identified a pattern of decreased levels of ether-bound phosphatidylcholines (PCs) that correlated with BMI and hypertension. A comparison between obese and nonobese twins revealed a more significant reduction in ether-bound lipids among obese twins compared to their nonobese counterparts [41]. This finding aligns with the observations in our study, where we noted reduced levels of all ether-bound lipids derived from phosphocholines and phosphoethanolamines. Notably, compounds like PC (O-34:3) were negatively correlated with hypertension, BMI, and insulin resistance, consistent with the results reported by Graessler et al. in 2009. [42].

Lipids bound by ether bonds are ubiquitous in most cell membranes, contributing to various essential functions such as mediating membrane dynamics and structure, acting as storage molecules for polyunsaturated fatty acids and lipid mediators, and serving as endogenous antioxidants [43]. Subjects with elevated triglycerides often exhibit low levels of plasmalogens [44]. Plasmalogens are particularly susceptible to oxidation due to the vinyl ether substituent at the sn-1 position of the glycerol main chain, making them preferential targets for oxidative damage [45]. Research indicates a decrease in plasmalogen concentration under conditions of oxidative stress, as observed in the oxidation of low-density lipoprotein (LDL) in atherosclerosis [46] and non-alcoholic steatohepatitis [47]. This decrease in plasmalogen levels found in our study might be related to oxidative stress, which also plays a significant role in the development of MetS [48].

Alterations in the metabolism of glycerophospholipids in various tissues have been documented in metabolic disorders such as obesity, insulin resistance, and atherosclerosis [49]. Variability in phosphatidylcholines (PCs), coupled with a reduction in lysophosphatidylcholines, has been linked to obesity [50], insulin resistance, and the development of type 2 diabetes [32]. In our study, we noted fluctuations in the levels of certain PCs. PC (33:2) and PC (33:1) exhibited higher concentrations in subjects with Metabolic Syndrome, showing associations with triglycerides and waist circumference and positive correlations with insulin and HOMA-IR. PC (33:1) also positively correlated with BMI, while PC (33:0), a discriminative factor between patient groups, displayed lower concentration.

Consistent with our findings, increased PC synthesis has been correlated with elevated diacylglycerol (DG) levels [51, 52]. Additionally, PC plays a vital role in the formation and secretion of very low-density lipoproteins and chylomicrons. The observed increase in PC levels in subjects with MetS might be associated with heightened synthesis of very low-density lipoprotein particles; this aligns with the proposed notion that increased hepatic synthesis of PC stimulates the production of very low-density lipoprotein particles [49]. Furthermore, the elevated plasma concentration of PCs has been linked to an unfavorable metabolic risk profile involving increased cholesterol and TG levels, given that around 65% of hepatic TG content appears to originate from hepatic PC [53]. However, some studies have correlated lower PC levels with cardiovascular risk factors, potentially linked to the fatty acid composition of PCs [54, 55].

LPCs are derived from the hydrolysis of PC by phospholipase A2 and can exert their biological functions by inducing cell division, releasing inflammatory factors, and promoting oxidative stress [56]. Alterations in plasma LPC levels have been reported in conditions like obesity and type 2 diabetes, with reduced LPC levels observed in healthy obese individuals and those with type 2 diabetes compared to their healthy counterparts [57]. In this study, we observed lower levels of LPC (20:4), LPC (18:1), and LPC (18:2) in the Metabolic Syndrome (MetS) group. However, when considering covariates such as sex and age, these differences lost statistical significance, suggesting that age and sex may play important roles in determining circulating LPC levels. Other studies have also identified differential LPC levels associated with age and sex [58].

Elevated phosphatidylinositols (PI) levels were also noted in our study, showing a correlation with triglyceride concentration. The relationship between PI levels and metabolic diseases has yielded varied findings in the existing literature. Animal models in experimental studies have suggested that inositol deficiency is linked to increased hepatic triglyceride accumulation [59] while supplementation with PI has been associated with improved insulin sensitivity, reduced TG concentration, and elevated high-density lipoprotein cholesterol (HDL-C) levels [60–62]. A study involving intraperitoneal streptozotocin found increased serum inositol and decreased hepatic inositol in rats with acute diabetes [63]. Meikle et al. reported a positive association between PI and phosphatidylserine levels in individuals with diabetes and prediabetes [64], and increases in myo-inositol levels have been documented in patients with severe heart failure [63, 65]. We observed a positive correlation of PI (32:1) with insulin and HOMA-IR, and PI (40:6) showed a positive correlation with insulin.

**Fig. 3:**
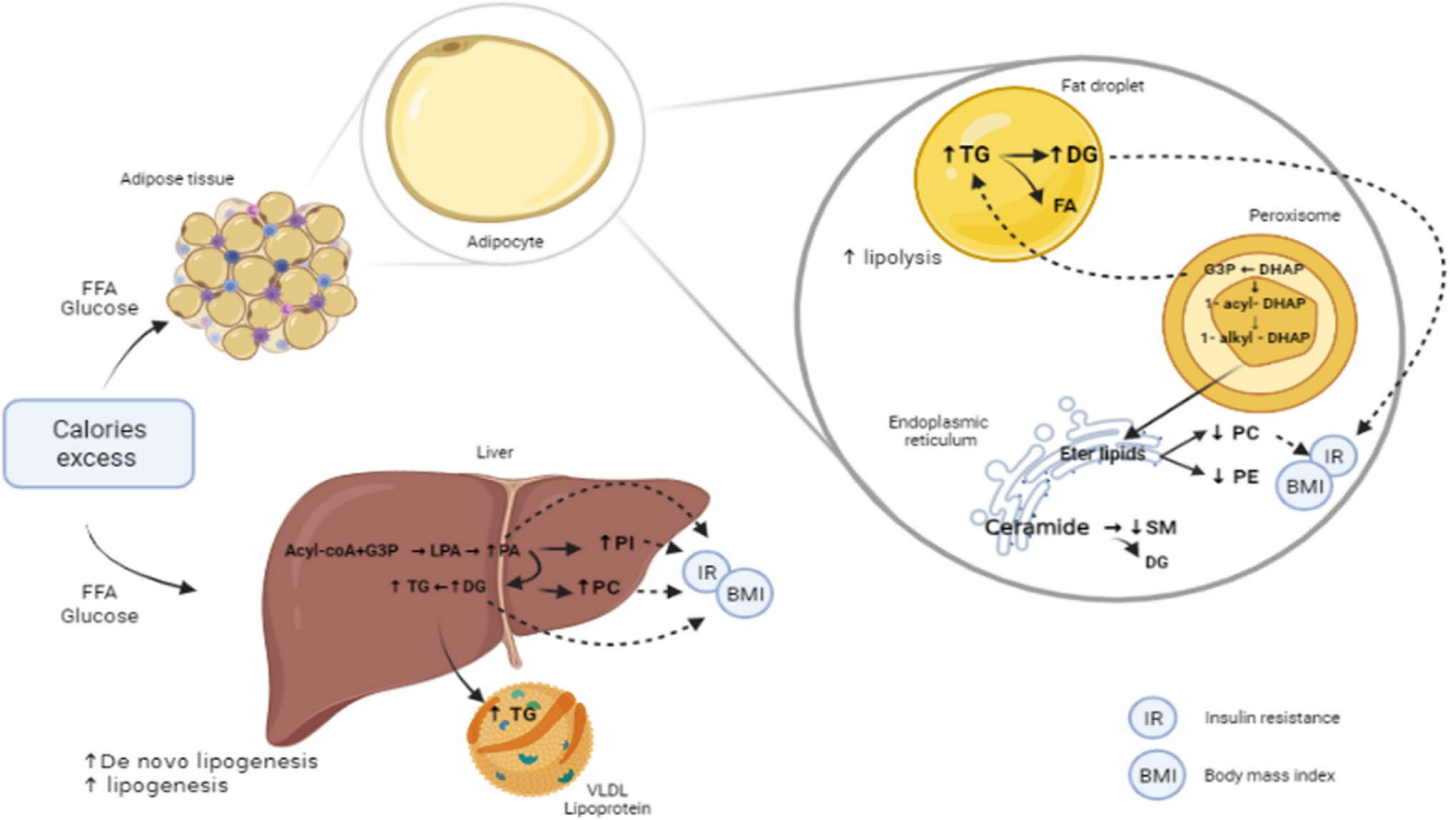
Lipid metabolic pathways associated with metabolic syndrome—MetS. Abdominal obesity and insulin resistance are the main mechanisms related to the alteration in lipid metabolism in the body. Plasma glucose and fatty acid levels are directly associated with dietary intake and stimulate lipogenesis pathways through various mechanisms. The subjects with MetS in this study presented an increase in the synthesis and degradation pathways of TG and DG. The increases in the TG and DG synthesis intermediates contribute to an alteration in the glycerophospholipids and sphingolipids pathways, generating a decrease in the synthesis and/or intermediates of these compounds. Abbreviations: **FFA:** Free Fatty acids; **TG:** triglycerides; **PI:** phosphatidylinositol; **PC:** phosphatidylcholine; **PE:** phosphatidylethanolamine; **G3P:** glycerol 3-phosphate; **LPA:** lysophosphatidic acid; **PA:** phosphatidic acid; **DG:** diacylglycerol; **VLDL:** very low-density lipoprotein; **SM:** sphingomyelin; **FA:** fatty acid

MetS is a complex disorder with a cluster of different factors that are not identical across individuals; however, our study has pinpointed pathways that significantly contribute to the elevation of triacylglycerol levels and increased waist circumference, particularly prevalent in our population (Fig. 3). A noteworthy discovery in our investigation is the identification of DG (34:2) and DG (38:5), two metabolites not only positively associated with MetS but also correlated with three key MetS components, higher insulin levels, HOMA-IR index, and BMI. These metabolites show potential as key molecules in understanding the pathways underlying MetS. Additionally, DG (42:6), positively associated with MetS and three of its components, represents a novel finding not previously reported as a significant lipid in individuals with MetS. This suggests that DG (42:6) could be a metabolite specifically relevant to the Latin-American population.

### Limitations and perspectives

The findings from this study highlight molecules such as TG, DG, and SM as potential markers for progression in individuals with MetS. Follow-up studies are essential to investigate whether this lipidomic profile is associated with the development of complications. A potential limitation of this study is the storage time of the samples. Although these samples were appropriately preserved, and other studies have demonstrated the stability of lipids in long-term storage. However, some less stable molecules may have degraded, limiting the presentation of a complete lipidomic profile. Longitudinal studies tracking patients and their lipidomic profiles, along with the development of complications, would provide valuable insights into the utility of these biomarkers for managing adults at risk.

## Conclusions

The lipidomic profile distinguishes adults with MetS from those without. Our results indicate that TG, DG, PC, and SM are crucial lipid classes reflective of the mechanisms underlying insulin resistance development through alterations in the synthesis and degradation pathways of glycerolipids and glycerophospholipids. Most of the lipids exhibiting differential levels in this study were correlated with serum insulin and HOMA-IR. These findings enhance our understanding of the metabolic pathways contributing to insulin resistance development and its physiopathology within the context of MetS. Furthermore, they suggest that an altered lipidomic profile, characterized by elevated levels of these molecules, may be associated with an inadequate progression of the disorder and an increased risk of developing complications.

### Supplementary Information

Below is the link to the electronic supplementary material.

Supplementary file1 (DOCX 76 kb)

## Data Availability

Derived data supporting the findings of this study are available from the corresponding author MM on request.
