# Supplementary material for: Lipidomic signatures in Colombian adults with metabolic syndrome
Source: J Diabetes Metab Disord. 2024 May 4;23(1):1279–92. doi: 10.1007/s40200-024-01423-5 (PMC11196482; doi:10.1007/s40200-024-01423-5)
Supplement: Supplementary file 1 — Supplementary file1 (DOCX 76 kb) [file 40200_2024_1423_MOESM1_ESM.docx]

Supplementary Material

**Table S1.** Differences in serum from lipidomic analysis (ESI +)

|  |  |  |  |  |  |  |  | **MetS vs HeS** | | |
| --- | --- | --- | --- | --- | --- | --- | --- | --- | --- | --- |
| **Compound** | **Formula** | **Mass** | **RT (min)** | **Mass Error (ppm)** | **Adduct** | **^a^ CV for QC (%)** | **CON** | **^b^ Change (%)** | **^c^ VIP** | **^d^ *p* value** |
| ***Fatty acids*** |  |  |  |  |  |  |  |  |  |  |
| FA (5:0) | C_5_H_10_O | 86.0732 | 4.39 | 5 | M+NH_4_ | 1.9 | Putative | -20 | 1.27776 | 0.0051 |
| Bile acids | C_24_H_38_O_4_ | 390.277 | 6.33 | 3 | M+H | 2.8 | Putative | 29 | 5.09008 | 1.00E-04 |
| FAHFA (41:5;0) | C_41_H_72_O_4_ | 628.5431 | 9.63 | 2 | M+H | 5.9 | Putative | 27 | - | 0.0466 |
| FAHFA (35:2;0) | C_35_H_66_O_4_ | 550.4961 | 11.49 | 2 | M+H | 19.5 | Putative | 41 | - | 0.0003 |
| FAHFA (39:4;0) | C_39_H_70_O_4_ | 602.5274 | 11.58 | 4 | M+H | 19.6 | Putative | 64 | - | 2.32E-05 |
| ***Glycerolipids*** |  |  |  |  |  |  |  |  |  |  |
| DG (38:7) | C_41_H_66_O_5_ | 638.491 | 10.35 | 2 | M+H | 5.3 | Putative | 35 | - | 0.0100006 |
| DG (32:1) | C_35_H_66_O_5_ | 566.491 | 10.78 | 2 | M+NH_4_ | 13.3 | Putative | 143 | - | 1.63E-05 |
| DG (36:5) | C_39_H_66_O_5_ | 614.491 | 10.93 | 2 | M+H | 9.4 | Putative | 65 | 1.00568 | 7.72E-07 |
| DG (36:3) | C_39_H_70_O_5_ | 618.5223 | 10.99 | 2 | M+NH_4_ | 12.9 | MS/MS | 46 | 1.08707 | 0.0142 |
| DG (38:6) | C_41_H_68_O_5_ | 640.5067 | 10.99 | 0 | M+H | 7.7 | Putative | 74 | - | 0.0011 |
| DG (34:3) | C_37_H_66_O_5_ | 590.491 | 11.49 | 2 | M+H | 14.2 | Putative | 62 | - | 2.75E-08 |
| DG (34:1) | C_37_H_70_O_5_ | 594.5223 | 11.54 | 2 | M+H | 6.4 | Putative | 114 | 2.10361 | 1.30E-07 |
| DG (34:2) | C_39_H_72_O_5_ | 620.538 | 11.58 | 2 | M+NH_4_ | 16.7 | Putative | 74 | 1.11505 | 3.02E-05 |
| DG (38:5) | C_41_H_70_O_5_ | 642.5223 | 11.58 | 1 | M+H | 18.0 | Putative | 105 | 1.13776 | 1.01E-08 |
| DG (42:6) | C_45_H_76_O_5_ | 696.5693 | 11.88 | 2 | M+NH_4_ | 5.3 | Putative | 135 | 1.20987 | 5.36E-10 |
| ***Glycerophospholipids*** |  |  |  |  |  |  |  |  |  |  |
| LPC (20:4) | C_28_H_50_NO_7_P | 543.3325 | 2.96 | 2 | M+H | 7.5 | MS/MS | -41 | 1.59858 | 0.0212* |
| LPC (15:0) | C_23_H_48_NO_7_P | 481.3168 | 2.97 | 2 | M+H | 2.3 | Putative | -21 |  | 0.0465 |
| LPC (18:2) | C_26_H_50_NO_7_P | 519.3325 | 3.05 | 2 | M+H | 6.4 | MS/MS | -40 | 3.19533 | 0.014 |
| LPC (18:1) | C_26_H_52_NO_7_P | 521.3481 | 4.18 | 3 | M+H | 4.0 | MS/MS | -31 | 2.15157 | 0.0397 |
| PC (O-16:1) | C_24_H_50_NO_6_P | 479.3376 | 4.32 | 2 | M+H | 3.3 | Putative | -35 | - | 0.0019 |
| PC (O-16:0) | C_24_H_52_NO_6_P | 481.3532 | 4.39 | 3 | M+H | 1.5 | MS/MS | -31 | - | 0.0044 |
| LPC (17:0) | C_25_H_52_NO_7_P | 509.3481 | 4.53 | 2 | M+H | 2.3 | MS/MS | -24 | - | 0.0135 |
| PC (O18:1) | C_26_H_54_NO_6_P | 507.3689 | 4.59 | 3 | M+H | 10.4 | Putative | -25 | - | 0.0122 |
| LPC(O-18:0) | C_26_H_56_NO_6_P | 509.3845 | 5.43 | 3 | M+H | 3.8 | Putative | -37 | - | 0.0007 |
| LPS(O-18:1) | C_24_H_48_NO_9_P | 525.3067 | 5.48 | 10 | M+H | 2.7 | Putative | -29 | - | 1.00E-04 |
| PC (O-36:4) | C_44_H_80_NO_9_P | 797.5571 | 6.34 | 0 | M+H | 3.9 | MS/MS | -24 | - | 0.0019 |
| PS (38:2) | C_44_H_82_NO_10_P | 815.5676 | 6.34 | 0 | M+H | 4.2 | Putative | -24 | - | 0.01 |
| PS(P-36:1) | C_42_H_80_NO_9_P | 773.5571 | 6.86 | 2 | M+H | 2.8 | Putative | -21 | 1.29767 | 0.0243 |
| PS(O-38:5) | C_44_H_78_NO_9_P | 795.5414 | 6.87 | 1 | M+H | 2.8 | Putative | -21 | - | 0.0196 |
| PS(O-40:6) | C_46_H_80_NO_9_P | 821.5571 | 7.39 | 1 | M+H | 2.0 | Putative | -21 | 1.05168 | 0.0078 |
| PE (39:3) | C_44_H_82_NO_8_P | 783.5778 | 7.42 | 1 | M+H | 7.6 | Putative | -23 | - | 0.0063 |
| PC (O-36:4) | C_44_H_80_NO_9_P | 797.5571 | 7.49 | 0 | M+H | 3.4 | MS/MS | -24 | - | 0.0135 |
| PS(P-38:1) | C_44_H_84_NO_9_P | 801.5884 | 7.71 | 1 | M+H | 7.1 | Putative | -20 | - | 0.0091 |
| PC(O-38:4) | C_46_H_84_NO_9_P | 825.5884 | 7.81 | 1 | M+H | 1.8 | Putative | -25 | - | 0.003 |
| PS(O-38:2) | C_44_H_84_NO_9_P | 801.5884 | 7.87 | 1 | M+H | 13.3 | Putative | -22 | - | 0.0063 |
| PS(O-40:4) | C_46_H_84_NO_9_P | 825.5884 | 8.36 | 1 | M+H | 17.3 | Putative | -20 | - | 0.0285 |
| PC (36:4) | C_44_H_80_NO_8_P | 781.5622 | 8.69 | 1 | M+H | 3.6 | MS/MS | -27 | 1.36996 | 0.003 |
| PC (32:1) | C_40_H_78_NO_8_P | 731.5465 | 9.11 | 2 | M+H | 1.8 | MS/MS | 21 | 2.93379 | - |
| PC(P-38:5) | C_46_H_82_NO_7_P | 791.5829 | 9.43 | 1 | M+H | 1.3 | Putative | -20 | - | 0.0237 |
| PC (P-36:4) | C_44_H_80_NO_7_P | 765.5672 | 9.52 | 2 | M+H | 2.3 | MS/MS | -23 | 1.299557 | 0.0293 |
| PC (O-36:4) | C_44_H_82_NO_7_P | 767.5829 | 9.68 | 2 | M+H | 3.3 | MS/MS | -24 | 1.38163 | 0.0122 |
| PC (P-34:2) | C_42_H_80_NO_7_P | 741.5672 | 9.7 | 1 | M+H | 2.6 | MS/MS | -39 | 1.81418 | 2.12E-05 |
| PC(P-36:5) | C_44_H_78_NO_7_P | 763.5516 | 9.71 | 0 | M+H | 4.2 | Putative | -33 | - | 0.0001 |
| PC(P-38:4) | C_46_H_84_NO_7_P | 793.5985 | 9.72 | 1 | M+H | 1.3 | MS/MS | -24 | 1.1232 | 0.0102 |
| PC (40:6) | C_48_H_84_NO_8_P | 833.5935 | 9.75 | 3 | M+H | 3.4 | MS/MS | 24 | 1.6463 | - |
| PE (O-37:2) | C_42_H_82_NO_7_P | 743.5829 | 9.85 | 1 | M+H | 4.4 | MS/MS | -38 | 1.25677 | 0.0001 |
| PC(O-36:3) | C_44_H_84_NO_7_P | 769.5985 | 9.89 | 1 | M+H | 5.5 | Putative | -38 | - | 0.0002 |
| PC (P-36:3) | C_44_H_82_NO_7_P | 767.5829 | 9.9 | 1 | M+H | 11.6 | MS/MS | -29 | - | 0.0042 |
| PC (38:3) | C_46_H_86_NO_8_P | 811.6091 | 10.33 | 2 | M+H | 2.7 | MS/MS | 32 | 2.92104 | - |
| PA (P-34:3) | C_37_H_67_O_7_P | 654.4624 | 10.35 | 2 | M+H | 4.8 | Putative | 43 | - | 0.0014 |
| PC (O-38:5) | C_46_H_84_NO_7_P | 793.5985 | 10.35 | 1 | M+H | 2.3 | MS/MS | -27 | - | 0.0039 |
| PE (O-37:2) | C_42_H_82_NO_7_P | 743.5829 | 10.35 | 1 | M+H | 2.9 | MS/MS | -25 | - | 0.0048 |
| PC (P-38:4) | C_46_H_84_NO_7_P | 793.5985 | 10.35 | 1 | M+H | 1.4 | MS/MS | -21 | - | 0.0102 |
| PE (38:1) | C_43_H_84_NO_8_P | 773.5935 | 10.36 | 1 | M+H | 2.7 | MS/MS | -22 | - | 0.0196 |
| PC (33:2) | C_41_H_78_NO_8_P | 743.5465 | 10.37 | 1 | M+H | 7.7 | MS/MS | 129 | - | 7.21E-05 |
| PE (O-35:0) | C_40_H_82_NO_7_P | 719.5829 | 10.43 | 1 | M+H | 5.1 | Putative | -27 | - | 0.0005 |
| PC (33:0) | C_41_H_80_NO_8_P | 745.5622 | 10.47 | 2 | M+H | 2.8 | MS/MS | -31 | 1.07478 | 0.0001 |
| PC (P-40:4) | C_48_H_88_NO_7_P | 821.6298 | 10.5 | 1 | M+H | 3.7 | Putative | -28 | - | 0.0003 |
| PC (P-38:3) | C_46_H_86_NO_7_P | 795.6142 | 10.51 | 2 | M+H | 6.9 | Putative | -26 | - | 0.0022 |
| PC(P-33:2) | C_41_H_78_NO_7_P | 727.5598 | 10.78 | 2 | M+H | 10.9 | Putative | -27 | - | 0.0025 |
| PC (P-33:2) | C_41_H_78_NO_7_P | 727.5516 | 10.8 | 2 | M+H | 10.7 | Putative | -35 | 3.3864 | 0.0001 |
| PA (P-32:2) | C_35_H_67_O_7_P | 630.4624 | 10.93 | 2 | M+H | 13.3 | Putative | 100 | - | 1.30E-07 |
| PC (33:1) | C_41_H_80_NO_8_P | 745.5622 | 10.97 | 2 | M+H | 16.2 | MS/MS | 42 | - | 0.005 |
| PA (O-34:3) | C_37_H_69_O_7_P | 656.4781 | 10.99 | 2 | M+H | 17.4 | Putative | 131 | - | 6.88E-06 |
| PC (P-42:4) | C_50_H_92_NO_7_P | 849.6611 | 11.23 | 2 | M+H | 5.7 | Putative | -32 | - | 0.0004 |
| PC (P-40:3) | C_48_H_90_NO_7_P | 823.6455 | 11.27 | 2 | M+H | 16.2 | Putative | -31 | - | 4.14E-05 |
| PC (P-42:2) | C_50_H_96_NO_7_P | 853.6924 | 11.31 | 4 | M+Na | 13.0 | Putative | -27 | - | 0.0137 |
| PC (36:3) | C_44_H_82_NO_8_P | 783.5778 | 11.73 | 1 | M+H | 13.0 | MS/MS | -23 | - | 0.0078 |
| PC (O-44:5) | C_52_H_96_NO_7_P | 877.6924 | 11.9 | 2 | M+H | 5.6 | Putative | -28 | - | 0.0004 |
| PE-NMe(44:6) | C_50_H_88_NO_8_P | 861.6248 | 9.01 | 6 | M+H | 2.5 | Putative | -26 | - | 0.0003 |
| PE (34:2) | C_39_H_74_NO_8_P | 715.5152 | 9.54 | 1 | M+H | 4.4 | Putative | 37 | - | 0.0349 |
| ***Sphingolipids*** |  |  |  |  |  |  |  |  |  |  |
| NeuAcalpha2-3Galbeta-Cer (d18:1/16:0) | C_51_H_94_N_2_O_16_ | 990.6603 | 3.84 | 7 | M+H | 3.2 | Putative | -24 | 1.60513 | - |
| SM (d18:1/16:0) | C_39_H_79_N_2_O_6_P | 702.5676 | 9.02 | 3 | M+H | 7.2 | MS/MS | -24 | 6.30935 | 0.0024 |
| SM (35:1) | C_40_H_81_N_2_O_6_P | 716.5832 | 9.31 | 2 | M+H | 3.4 | Putative | -31 | - | 0.0009 |
| SM (34:0) | C_39_H_81_N_2_O_6_P | 704.5832 | 9.37 | 1 | M+H | 2.6 | MS/MS | -30 | 1.05003 | 0.0026 |
| SM (40:3) | C_45_H_87_N_2_O_6_P | 782.6302 | 10.03 | 0 | M+H | 2.2 | Putative | -21 | - | 0.0078426 |
| SM(d40:1) | C_45_H_91_N_2_O_6_P | 786.6615 | 10.12 | 0 | M+Na | 3.8 | Putative | -31 | - | 0.0011 |
| SM (40:2) | C_45_H_89_N_2_O_6_P | 784.6458 | 10.72 | 1 | M+H | 6.7 | Putative | -24 | - | 0.0196 |
| SM (38:1) | C_43_H_87_N_2_O_6_P | 758.6302 | 10.76 | 2 | M+H | 7.6 | MS/MS | -20 | 1.57877 | 0.0336 |
| SM (40:2) | C_45_H_89_N_2_O_6_P | 784.6458 | 10.85 | 2 | M+H | 15.2 | MS/MS | -33 | 2.48024 | 0.0004 |
| SM (41:2) | C_46_H_91_N_2_O_6_P | 798.6615 | 11.09 | 1 | M+H | 9.8 | MS/MS | -33 | 1.32051 | 0.0002 |
| SM (39:1) | C_44_H_89_N_2_O_6_P | 772.6458 | 11.15 | 1 | M+H | 7.7 | MS/MS | -33 | 1.68548 | 0.00044 |
| SM (41:1) | C_46_H_91_N_2_O_6_P | 798.6615 | 11.24 | 2 | M+H | 9.2 | Putative | -40 | 2.60912 | 1.26E-05 |
| SM (41:0) | C_46_H_93_N_2_O_6_P | 800.6771 | 11.85 | 2 | M+H | 5.3 | MS/MS | -26 | 1.81845 | 0.0075 |
| SM(d33:0) | C_38_H_77_N_2_O_6_P | 688.5519 | 8.56 | 3 | M+H | 14.9 | Putative | -24 | 1.01169 | 0.0078 |
| SM(d34:2) | C_39_H_77_N_2_O_6_P | 700.5519 | 8.71 | 2 | M+H | 5.8 | Putative | -40 | - | 0.0004 |
| ***Sterol lipids*** |  |  |  |  |  |  |  |  |  |  |
| Cholesteryl 11-hydroperoxy-eicosatetraenoate | C_47_H_76_O_4_ | 704.5744 | 11.2 | 1 | M+NH_4_ | 13.1 | Putative | -30 | - | 0.0009 |
| ***Prenol lipids*** |  |  |  |  |  |  |  |  |  |  |
| Fucoxanthinol eicosapentaenoate | C_60_H_84_O_6_ | 900.6268 | 10.81 | 6 | M+H | 7.0 | Putative | -25 | - | 0.0022 |
| Fucoxanthinol stearidonate | C_58_H_82_O_6_ | 874.6111 | 10.85 | 5 | M+H | 5.2 | Putative | -23 | - | 0.0073 |

^a^ CV for QC (%): CV obtained for the same compound within the set of quality control samples. ^b^ CON: lipid confirmation by MS/MS. ^c^ Change: percentage change in abundances. calculated as MetS/HeS. The sign indicates the change direction. ^d^ VIP: values with Estimation of Jack-Knife confidence interval without including the 0 - confidence level: 95%. ^e^ p values corrected by Benjamin Hochberg (FDR correction). * Significant lipids after adjustment for sex and age covariates. Abbreviations: FA: Fatty acids, FAHFA: Fatty Acid ester of Hydroxyl Fatty Acid, DG: Diacylglycerol, LPC: Lysophosphatidylcholines, PC: Phosphatidylcholine, LPS: Lipopolysaccharide, PS: Phosphatidylserine, PE: Phosphatidylethanolamine, PA: Phosphatidic acid, SM: Sphingomyelin.

**Table S2.** Differences in serum from lipidomic analysis (ESI -)

|  |  |  |  |  |  |  |  | **MetS vs HeS** | | |  |
| --- | --- | --- | --- | --- | --- | --- | --- | --- | --- | --- | --- |
| **Compound** | **Formula** | **Mass** | **RT (min)** | **Mass Error (ppm)** | **Adduct** | **^a^ CV for QC (%)** | **CON** | **^b^ Change (%)** | **^c^ VIP** | **^d^ *p* value** | |
| ***Fatty acids*** |  |  |  |  |  |  |  |  |  |  | |
| Isobutyl furanpropionate//oxo-undeca dienoic acid | C_11_H_16_O_3_ | 196.1099 | 1.22 | 2 | M-H- | 4.3 | Putative | 45 | - | 0.0235* | |
| Hydroxy-arachidonic acid | C_20_H_32_O_3_ | 320.2351 | 4.7 | 1 | M-H- | 3.0 | MS/MS | -22 | 1.75704 | 0.0318* | |
| Hexacosatrienoic acid//Eicosatrienyl methylvalerate | C_26_H_46_O_2_ | 390.3498 | 8.68 | 0 | M+CHO_2_- | 4.3 | Putative | -28 | - | 0.0361* | |
| ***Glycerolipids*** |  |  |  |  |  |  |  |  |  |  | |
| DG (44:12) | C_47_H_68_O_5_ | 712.5067 | 9.33 | 8 | M-H-H_2_O | 5.5 | Putative | 60 | - | 0.0112* | |
| DG (44:11) | C_47_H_70_O_5_ | 714.5223 | 10.13 | 9 | M-H-H_2_O | 3.5 | Putative | 73 | 1.66118 | 0.019 | |
| TG (59:13) | C_62_H_94_O_6_ | 934.705 | 11.4 | 5 | M+CHO_2_- | 4.9 | Putative | 39 | - | 0.0188* | |
| TG (61:14) | C_64_H_96_O_6_ | 960.7207 | 11.4 | 5 | M+CHO_2_- | 3.4 | Putative | 72 | 1.49631 | 0.019 | |
| ***Glycerophospholipids*** |  |  |  |  |  |  |  |  |  |  | |
| PI (36:3) | C_45_H_81_O_13_P | 860.5415 | 6.69 | 3 | M-H- | 8.7 | Putative | -20 | - | 0.02995* | |
| PI (40:4) | C_49_H_87_O_13_P | 914.5884 | 7.7 | 3 | M-H- | 3.2 | Putative | -24 | 1.58543 | 0.01795* | |
| PI (40:3) | C_49_H_89_O_13_P | 916.6041 | 7.8 | 3 | M-H- | 3.1 | MS/MS | -21 | 3.91535 | 0.01210* | |
| PI (36:4) | C_45_H_79_O_13_P | 858.5258 | 10.51 | 0 | M-H- | 3.4 | Putative | 29 | 1.2599 | 0.01437* | |
| PI (32:1) | C_41_H_77_O_13_P | 808.5102 | 10.52 | 0 | M-H- | 3.8 | Putative | 57 |  | 0.019 | |
| PI (40:6) | C_49_H_83_O_13_P | 910.5571 | 11.17 | 1 | M-H- | 3.9 | Putative | 21 |  | 0.00880* | |
| PS (35:0) | C_41_H_80_NO_10_P | 777.552 | 11.31 | 0 | M-H- | 3.3 | MS/MS | 29 | 1.77963 | 0.01216* | |
| PI (40:5) | C_49_H_85_O_13_P | 912.5728 | 11.41 | 0 | M-H- | 7.3 | Putative | 52 | - | 0.01289* | |
| PC (31:2) | C_39_H_74_NO_8_P | 715.5152 | 11.67 | 0 | M-H- | 5.1 | MS/MS | 34 | - | 0.01173* | |
| PI (38:3) | C_47_H_85_O_13_P | 888.5728 | 11.79 | 0 | M-H- | 3.2 | Putative | 32 | - | 0.036 | |
| ***Sphingolipids*** |  |  |  |  |  |  |  |  |  |  | |
| NeuAcalpha2-3Galbeta1-4Glcbeta-Cer(d18:1/20:0) | C_61_H_112_N_2_O_21_ | 1189.7563 | 9.06 | 1 | M-H-H_2_O | 3.5 | Putative | -25 |  | 0.01379* | |
| ***Esterol lipids*** |  |  |  |  |  |  |  |  |  |  | |
| Vitamin D2 sulfur dioxide adducts | C_28_H_44_O_3_S | 460.3011 | 6.74 | 6 | M-H- | 6.3 | Putative | -46 | 4.7499 | - | |
| Trioxocholenoic Acid | C_24_H_32_O_5_ | 400.225 | 4.69 | 7 | M+Cl- | 4.7 | Putative | -22 | - | 0.032 | |
| ***Prenol lipids*** |  |  |  |  |  |  |  |  |  |  | |
| Etretinate | C_23_H_30_O_3_ | 354.2195 | 4.73 | 1 | M-H- | 5.8 | Putative | -48 | 2.72468 | - | |

^a^ CV for QC (%): CV obtained for the same compound within the set of quality control samples. ^b^ CON: lipid confirmation by MS/MS. ^c^ Change: percentage change in abundances. calculated as MetS/HeS. The sign indicates the change direction. ^d^ VIP: values with Estimation of Jack-Knife confidence interval without including the 0 - confidence level: 95%. ^e^ p values corrected by Benjamin Hochberg (FDR correction). * Significant lipids after adjustment for sex and age covariates. Abbreviations: DG: Diacylglycerol, TG: Triglycerides, PI: Phosphatidylinositol, PS: Phosphatidylserine, PC: Phosphatidylcholine,

**Table S3.** Lipids with Differential Expression within Metabolic Syndrome components

| **Lipid class** | **Lipid** | **^a^ Change** | **^b^ VIP** | **TG** | ^c^ **Waist** | **HDL** | **SBP** | **Glucose** |
| --- | --- | --- | --- | --- | --- | --- | --- | --- |
| Fatty acids | Bile acids | 29 | 5.09008 | 0.00017672 | 9.0715E-05 | - | - | - |
|  | FAHFA (35:2;0) | 41 | - | 0.00014902 | 0.00015895 | - | - | - |
|  | FAHFA (39:4;0) | 64 | - | 1.94E-07 | 8.0326E-05 | 0.00012611 | - | - |
| Glycerolipids | DG (32:1) | 143 | - | 6.59E-09 | 5.70E-06 | 0.00017141 | - | - |
|  | DG (34:1) | 114 | 2.10361 | 9.46E-11 | 8.15E-08 | 9.03E-05 | - | - |
|  | DG (34:2) | 74 | 1.11505 | 2.11E-08 | 1.5171E-05 | 2.6751E-05 | - | - |
|  | DG (34:3) | 62 | - | 1.19E-10 | 3.28E-10 | 1.30E-04 | 2.45E-04 | 3.25E-05 |
|  | DG (36:3) | 46 | 1.08707 | 3.38E-05 | 2.24E-04 | - | - | - |
|  | DG (38:5) | 105 | 1.13776 | 6.30E-14 | 2.06E-08 | 1.96E-08 | - | - |
|  | DG (38:7) | 35 | - | 1.16E-04 | - | - | - | - |
|  | DG (42:6) | 135 | 1.20987 | 1.17E-07 | 3.28E-05 | 3.94E-04 | 1.25E-04 | - |
|  | DG (36:5) | 65 | 1.00568 | 1.64E-10 | 4.66E-07 | - | - | - |
|  | DG (38:6) | 74 | - | 2.86E-06 | 4.81E-04 | - | - | - |
|  | DG (44:11) | 73 | 1.66118 | 5.38E-05 | - | - | 5.46E-05 | - |
|  | DG (44:12) | 60 | - | - | - | - | 0.0002263 | - |
|  | TG (61:14) | 72 | 1.49631 | 3.88E-05 | - | - | 2.08E-05 | - |
| Glycerophospholipids | LPS (O-18:1) | -29 | - | 6.0519E-05 | 0.0004527 | - | - | - |
|  | PA (O-34:3) | 131 | - | 4.24E-09 | 1.3749E-05 | 0.000472 | - | - |
|  | PA (P-32:2) | 100 | - | 1.46E-10 | 1.08E-06 | - | 1.23E-04 | - |
|  | PA (P-34:3) | 43 | - | 1.04E-05 | - | - |  | - |
|  | PC (33:0) | -31 | 1.07478 | - | 1.31E-04 | - |  | - |
|  | PC (33:1) | 42 | - | 1.09E-06 | - | - |  | - |
|  | PC (33:2) | 129 | - | 7.32E-08 | 6.28E-05 | - | 0.000124 | - |
|  | PC (P-34:2) | -39 | 1.81418 | - | 2.03E-04 | - | - | - |
|  | PC (O-36:4) | -29 | - | - | 1.76E-04 | - | - | - |
|  | PC (O-44:5) | -28 | - | - | 3.96E-04 | - | - | - |
|  | PC (P-33:2) | -35 | 3.3864 | - | 0.00017504 | - | - | - |
|  | PC (P-40:3) | -31 | - | - | 2.93E-04 | - | - | - |
|  | PC (P-42:4) | -27 | - | 4.77E-04 | 1.91E-05 | - | - | - |
|  | PC(P-36:5) | -33 | - | - | 4.64E-04 | - | - | - |
|  | PE (O-35:0) | -27 | - | - | 2.15E-04 | - | - | - |
|  | PI (32:1) | 57 |  | 1.86E-04 | - | - | - | - |
|  | PI (36:4) | 29 | 1.2599 | 3.82E-04 | - | - | - | - |
|  | PI (38:3) | 32 | - | 4.15E-05 | - | - | - | - |
|  | PI (40:5) | 52 | - | 2.26E-04 | - | - | - | - |
|  | PI (40:6) | 21 |  | 1.13E-05 | - | - | - | - |
| Sphingolipids | SM (41:1) | -40 | 2.60912 | - | 2.73E-04 | - | - | - |
|  | SM (d34:2) | -40 | - | - | 7.993E-05 | - | - | - |
|  | SM (d40:1) | -31 | - | - | 4.64E-04 | - | - | - |
| The values expressed in the table correspond to the p values corrected by Bonferroni correction for each of the lipids concerning the components of the metabolic syndrome obtained by the ANCOVA test and adjusted for age and sex covariates. ^a^ Change: percentage of change in abundances, calculated as MetS/control, the sign indicates the direction of change. ^b^ VIP: values with Estimation of Jack-Knife confidence interval without including the 0 - confidence level: 95%. Abbreviations: TG: triglycerides, HDL: High-Density Lipoproteins, ^c^ Waist: Waist circumference, SBP: Systolic blood pressure, FAHFA: Fatty Acid ester of Hydroxyl Fatty Acid, DG: Diacylglycerol, TG: Triglycerides, LPS: Lipopolysaccharide, PA: Phosphatidic acid, PC: Phosphatidylcholine, PE: Phosphatidylethanolamine PI: Phosphatidylinositol, SM: Sphingomyelin. | | | | | | | | |
